# Biofilm Formation by *Staphylococcus epidermidis* on Foldable and Rigid Intraocular Lenses

**DOI:** 10.5812/jjm.10020

**Published:** 2014-05-01

**Authors:** Bibi Sedigheh Fazly Bazzaz, Monireh Jalalzadeh, Maryam Sanati, Syamak Zarei-Ghanavati, Bahman Khameneh

**Affiliations:** 1 Biotechnology Research Centre, School of Pharmacy, Mashhad University of Medical Sciences, Mashhad, IR Iran; 2 School of Medicine, Mashhad University of Medical Sciences, Mashhad, IR Iran; 3 Department of Food and Drug Control, School of Pharmacy, Mashhad University of Medical Sciences, Mashhad, IR Iran

**Keywords:** Biofilms, Endophthalmitis, Lenses, Intraocular, *Staphylococcus epidermidis*

## Abstract

**Background:**

Biofilm formation of *Staphylococcus epidermidis* is a major etiological factor of inducing device-related infections.

**Objectives:**

The ability of biofilm formation by the *S. epidermidis* was assessed *in vitro* on two brands of foldable (hydrophilic) and two brands of rigid (hydrophobic) intraocular lens materials in order to investigate the role of lens material in postoperative endophthalmitis.

**Materials and Methods:**

To ensure reproducibility of biofilm formation on intraocular lenses, two strains of *S. epidermidis* and three quantification methods were performed. The *S. epidermidis* strains, DSMZ3270 (biofilm-producer) and ATCC12228 (non-biofilm-producer) were applied. Organisms were cultivated on disks of different brands of foldable hydrophilic Intra Ocular Lens (IOL) made of acrylic (Didar, Iran; (A) and Omni, India; (B)), and rigid hydrophobic IOL made of polymethyl methacrylate (PMMA; Didar, Iran; (C) and Hexavision, France; (D)). Biofilms were stained with crystal violet (CV) dye, which is an index of biofilm formation. The bacterial population was counted after biofilm homogenization. Scanning electron microscopy (SEM) was performed to examine the extent of biofilm formation.

**Results:**

Adherence of DSMZ3270 strain on both types of foldable and rigid IOLs, was significantly more than ATCC12228 (P < 0.001-0.05 and, P < 0.01-0.05, respectively). The bacterial populations between the lenses were significantly different (P < 0.05). Subsequent studies demonstrated significant differences between brands of foldable and PMMA IOLs. According to statistical analyses the incubation time influenced the biofilm formation on both types of IOLs which meant that by increasing incubation time, the biofilm formation increased. According to the SEM pictures, biofilm seems to be lysed at 72 hours.

**Conclusions:**

These data demonstrated that the attachment of bacteria to hydrophilic acrylic IOLs was more than hydrophobic PMMA ones independent of the brand. According to these results the bacterial strain might have more hydrophilic properties. Augmenting the biomass of biofilm by passing of time demonstrated the key role of time in biofilm formation on the IOL surfaces. The differences between IOL brands in the biofilm formation indicated the influence of design parameters for IOLs.

## 1. Background

Several approaches such as development of microsurgery and viscosurgery or intraocular lens (IOL) insertion were developed to succeed in cataract extraction among them, IOL implementation can have pronounced advantages. The advantages are lost if any microbial contamination occurs. Microbial adsorption, adhesion, and colonization could cause biofilm formation on abiotic surfaces such as IOL (1, 2). Thus biofilm formation is considered as one of the most serious problems of cataract surgery. Therefore, development of an effective IOL with appropriate properties would be desirable as a means to reduce the risk of ocular complications (3).

Nowadays, the most commonly used IOL materials are poly (methyl methacrylate) (PMMA), acrylic, hydrogel, and silicone. Since a high variety of materials have been used to form IOLs, different properties were predicted. These variations affect the IOL features such as foldable or rigid and/or hydrophilic or hydrophobic properties. A recent review showed that rigid spherical PMMA IOLs were the most frequently used IOLs (4). It is also noteworthy that PMMA IOLs are the first choice of rigid material (5). Despite all potential advantages, there are certain unpredictable possible risks that may occur after the implant surgery. 

Microbial infection and inflammation are regarded as the most important surgical risks. In the field of inflammation, postoperative endophthalmitis is still known as one of the most serious and damaging issues rising in intraocular surgery. In most cases, it leads to massive and long-lasting deterioration of visual acuity (1). It is reported that the incidence of endophthalmitis after cataract extraction and IOL implantation is 0.1% to 0.3% in the Western countries (6). As mentioned previously, microbial infections cause serious problems (7). Most of the pathogens inserted into the eye during the surgery are related to the microbial flora of the external ocular. *Staphylococcus epidermidis*, Gram-positive coagulase-negative cocci, is one of human normal floras. 

This microorganism has turned into a serious leading opportunistic pathogen of nosocomial infections. A major factor that attributes to *S. epidermidis* pathogenicity in device-associated infections is formation of biofilm (8). Biofilm formation is an underlying strategy used by some bacteria to survive in the natural environments (9, 10). Considering the different features of bacterial biofilms in comparison with planktonic counterparts, their treatment is much more difficult. It is also noteworthy that the bacteria in biofilms are more resistant to antiseptics, antibiotics, and host defenses (2, 11). The respective increase of the biofilm resistance to the treatment lies in the fact that they could form complicated structures (12). It was demonstrated that biofilm exopolysaccharides as complicated structures help the bacteria to firmly adhere to the inert layer.

Although *S. epidermidis* and other coagulase negative Staphylococci are usually the responsible microorganisms in the majority of implanted foreign material infections, the proportion alters depending on the type of infection and the organ surveyed. *S. epidermidis* is responsible for about 60% of cases of the most acute endophthalmitis (2). *S. epidermidis* is also the common organism of chronic endophthalmitis. In general, it is believed to be the most frequent microorganism in postoperative cataract extraction and IOL implantation. This bacteria which normally originates from microflora engendered by the patient’s eyelids and conjunctiva, is the predominant causative organism (13). 

*S. epidermidis* can enter the eye through the incision sites throughout the eye surgery. Then, it can adhere to the IOL in both the anterior chamber and intraocular tissues. This is regarded as an overarching issue, since the responsible microorganisms colonized on the surface of the implanted materials can produce an extracellular polysaccharide substance which is a biofilm (slime). Since different materials and designs parameters have been applied to produce IOLs, the relative adherence capacities of bacteria to the IOLs are different.

## 2. Objectives

In the previous studies, bacterial adhesion on the IOLs was investigated. Since bacterial adhesion depends on some properties of IOLs such as hydrophobicity and/or rigidity, the current study aimed to assess bacterial adhesion on the four different brands. These IOLs were selected from foldable hydrophilic and rigid hydrophobic IOLs. 

## 3. Materials and Methods

### 3.1. Intraocular Lenses

The test was carried out on two different types of posterior chamber IOLs, which included foldable and rigid materials, with an optic diameter of 6 mm. Disks of foldable hydrophilic IOL made of acrylic (Didar, Iran; (A) and Omni, India; (B)), and rigid hydrophobic IOL made of polymethylmethacrylate (PMMA; Didar, Iran; (C) and Hexavision, France; (D)) were evaluated. 

### 3.2. Bacteria and Media

The *S. epidermidis* strains DSMZ3270 (DSMZ Cloning, Germany) (biofilm-producer) and ATCC12228 (American Type Culture Collection) (non-biofilm-producer) were used. Stock cultures of bacteria were frozen at -75°C in brain–heart infusion broth medium (BHI, Biomrieux, France) containing 25% glycerol. Before each attempt, small quantities of the bacterial culture were subcultured in the BHI broth overnight at 37°C in order to make sure about the purity and viability. DSMZ3270 is an adherent, slime-producing strain. All *S. epidermidis* strains were cultivated in trypticase soy broth (TSB, Merck, Germany) supplemented with 0.25% glucose.

### 3.3. IOL Biofilm Assay Using Crystal Violet

The ability of *S. epidermidis* to create biofilms on abiotic surfaces was quantified essentially as explained previously (14). Briefly, an overnight culture of *S. epidermidis* was grown in TSB with 0.25% glucose at 37°C for 18-20hours. The IOLs were attached to the bottom of a 96-well polystyrene microtiter plate (Orange Scientific, Belgium) through its plastic side struts (haptics) with the help of special forceps. The bacterial cultures were diluted 1:40 in TSB containing 0.25% glucose and then the wells were filled with 200 μL of diluted culture and incubated at 37°C. After an incubation period of 24 and 72 hours of biofilms cultivation on disk, each IOL was rinsed three times with 200 μL phosphate-buffered saline (PBS), dried, and stained with crystal violet (1%) for 15 minutes. The IOLs were rinsed again with PBS to evacuate unbound biofilm. To ensure about its sterilization, the liquid was seeded from the last wash-up. In order to solubilize, bound crystal violet 200 μL of ethanol-acetone (80:20, vol/vol) was added to each well. The optical density at 600nm was determined with a microplate reader (Awareness, UK). Each experiment was performed in five replicate wells.

### 3.4. Bacterial Population Enumeration of IOL

After the aforementioned process of incubation during which biofilms were cultivated on disk, each IOL was washed three times gently with PBS and was transferred to a sterile 1.5 mL microtube (LockFit; Treff, Degersheim, Switzerland) containing 300 μL of 1 mm diameter sterile glass beads (SGMT No. 001; USA) in 1 mL of PBS. Then the tubes were vortexed (Velp, Germany) for 1.5 minutes at 2500 rpm in order to separate the cells from its biofilm matrix. This regimen has been efficient by removing all the adherent bacteria with maximum number of colony-forming units (CFU) without affecting their viability (11). After vortexing, the extracted bacteria were enumerated using agar dilution plating technique. To perform it, 10 serial fold dilutions (1/10, 1/100, and 1/1000) were made from each sample containing the dislodged bacteria and 10 microliters were seeded to calculate an accurate count of the bacteria adhered to the lenses. Each experiment was performed in triplicate.

### 3.5. Scanning Electron Microscopy

After the foregoing incubation, each IOL was gently washed three times with PBS. First the IOLs were fixed with 2.5% (wt/vol) glutaraldehyde in a filter-sterilized phosphate buffer (0.1 M, pH 7.4) at room temperature for two hours and then rinsed three times for 15 minutes in sodium cacodylate buffer (0.1 M). Next, a second fixation step was performed for one hour with osmium tetroxide (1%wt/vol) in a sodium cacodylate buffer (0.1 M). Quick rinse in distilled water was the next step of preparation. The fixed lenses were then dehydrated in successive ethanol-water mixtures by escalating the percentage of ethanol (50%, 70%, 80%, and 95% by volume) for seven minutes each, and then two times in pure ethanol for 15 minutes. They were put into an ethanol bath in order for the process of evaporation to take place. The dried samples were attached to the metal holders with double-sided adhesive tape and ultimately coated with platinum and palladium in an evaporator. Observations were performed at 15kV with a scanning electron microscope (model LEO, Germany). From the optic surface of each sample, three fields of view were randomly selected with a magnification from × 1000. Each experiment was performed in triplicate.

### 3.6. Statistical Analysis

Adhesion data collected from every IOL were compared with a one way ANOVA (preliminary tests). Parametric tests (Tukey test), which allow mean comparisons (biofilm-producer versus non biofilm-producer strain, different incubation times and different IOLs) were carried out.

## 4. Results

### 4.1. Biofilm Formation on IOLs Using Crystal Violet

**Figure 1.**
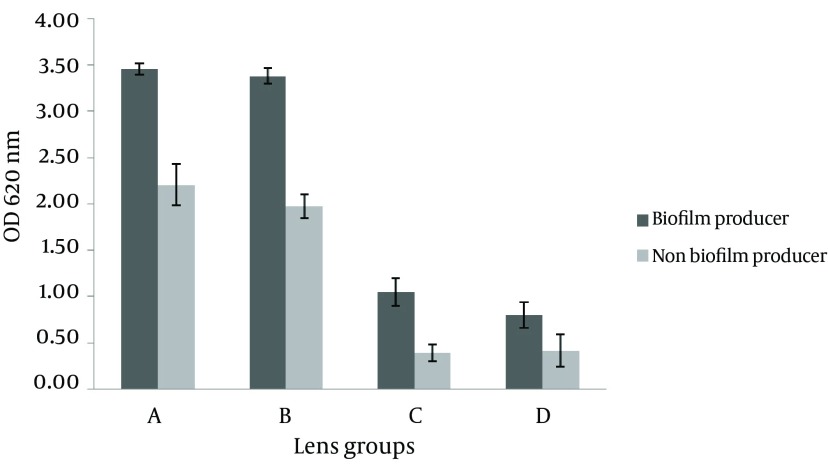
The Mean Optical Density of Each Brand of IOLs Which Stained With 1% Crystal Violet After Exposure to Bacterial Strains Bars and error bars represent the mean ± SD of results in five replicate experiments. Bars of A and B refer to hydrophilic IOLs; C and D bars illustrate hydrophobic IOLs.

**Figure 2.**
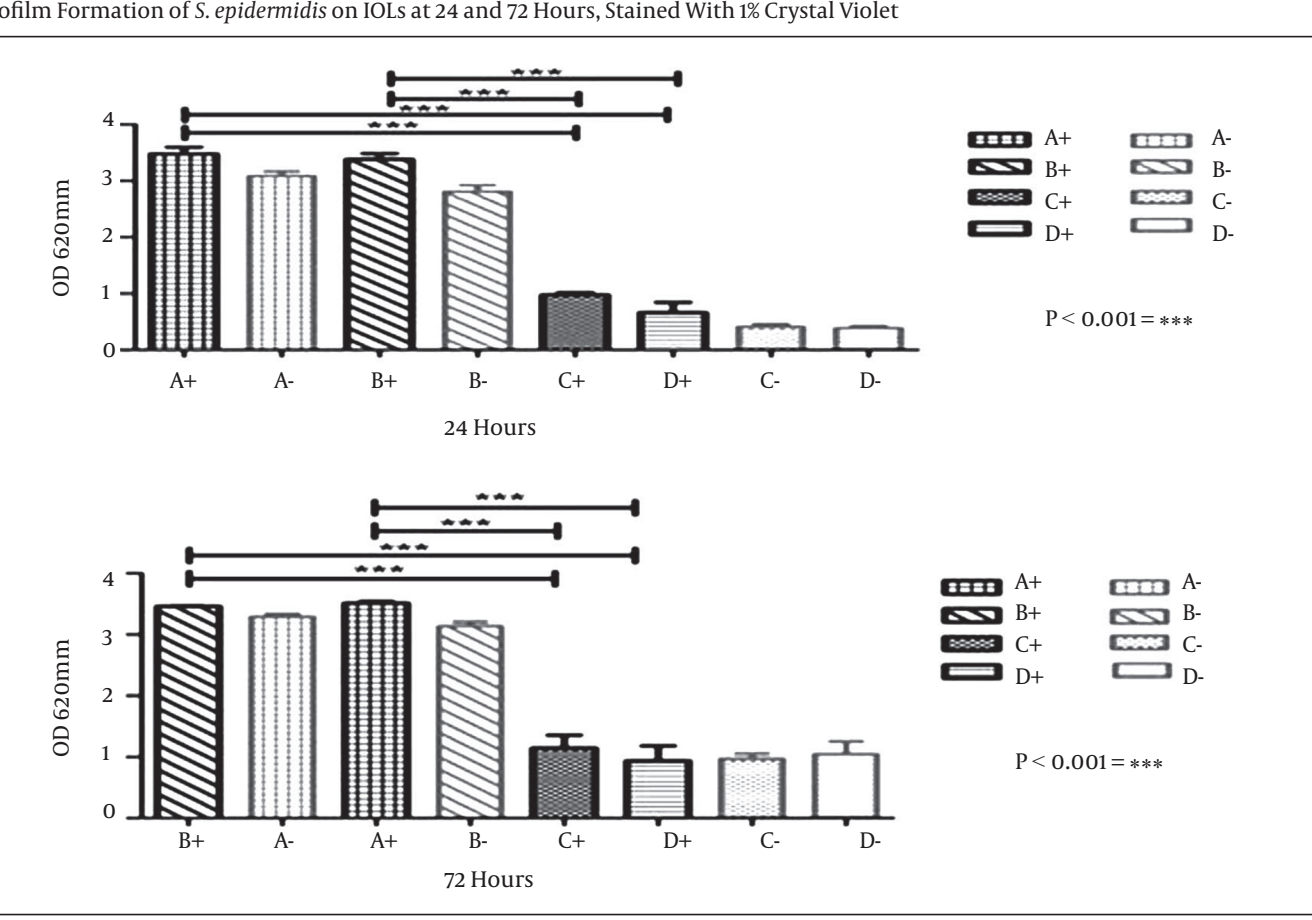
Biofilm Formation of *S. epidermidis* on IOLs at 24 and 72 Hours, Stained With 1% Crystal Violet Results are illustrated with the ATCC12228 (non-biofilm) and DSMZ3270 (biofilm producer) strains at 24 and 72 hours. Bars and error bars represent the mean ± SD of results in five replicate experiments. "+" and "-" symbols referred to the biofilm-producer and non-producer *S. epidermidis* strains, respectively. Bars of A and B refer to hydrophilic IOLs; C and D bars illustrate hydrophobic IOLs.

*S. epidermidis* biofilm formation on each brand of IOL initiated with nearly 106 CFU/mL of strains ATCC12228 and DSMZ3270. The biofilm was afterwards detected by crystal violet staining after 24 and 72 hours of incubation. The optical density of the biomass of DSMZ3270 was generally greater than that of ATCC12228. As depicted in Figures 1 and 2, there was a significant difference between the biofilm formation in hydrophobic acrylic and hydrophilic PMMA lenses (P < 0.05). According to the statistical analyses, there were significant differences between brands of hydrophilic foldable IOLs (P < 0.05). Considering that in rigid hydrophobic IOLs there were no significant differences between brands (Data not shown), the effect of time on biofilm formation was analyzed. These data demonstrated that by increasing incubation time from 24 to 72 hours the biomasses on both types of IOLs increased (P < 0.05). According to these data biofilm formation on IOLs showed significant differences between 24 and 72 hours (P < 0.05, Data not shown).

### 4.2. Bacterial Population Enumeration of IOLs

**Figure 3.**
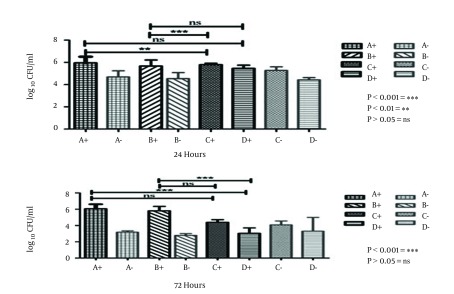
Quantification of Adherent Bacteria on IOLs Results are shown with the *S. epidermidis* ATCC12228 (2) and DSMZ3270 (+) strains at 24 and 72 hours. "+" and "-" symbols referred to the biofilm-producer and non-producer *S. epidermidis* strains, respectively. Bars and error bars represent the mean ± SD of results in triplicate experiments (n = 5; *P < 0.05, ns = non- significant). Bars of A and B refer to hydrophilic IOLs; C and D bars illustrate hydrophobic IOLs.

Homogenization of the biofilm on the IOL was determined through quantitative counting. As illustrated in Figure 3, there were no significant differences in the number of adherent bacteria between A brand of hydrophilic and D brand of hydrophobic IOLs and also B brand of hydrophilic and D brand of hydrophobic at 24 hours (P > 0.05). Hydrophilic acrylic IOLs at 72 hours, there were no significant differences between A brand of hydrophilic and C brand of hydrophobic IOLs and also B brand of hydrophilic and C brand of hydrophobic. However, significant differences were observed in hydrophobic ones (P < 0.05).

**Figure 4.**
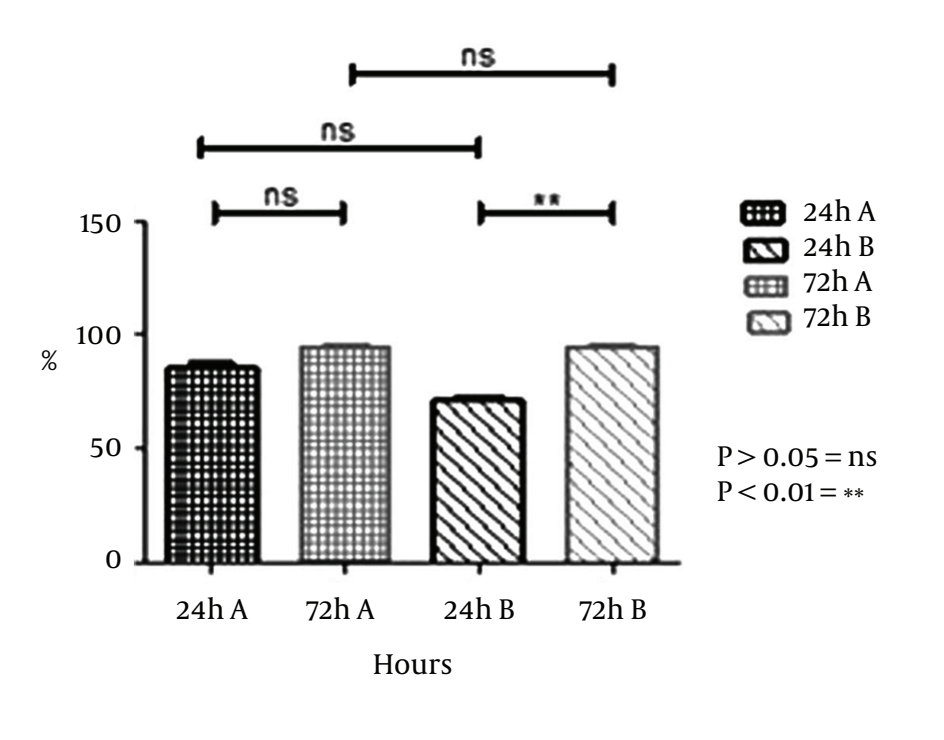
Ratio of Positive Field in SEM Picture on Different Brands of Hydrophilic IOLs With the *S. epidermidis* DSMZ3270 Strain Bars and error bars represent the mean ± SD of results in triplicate experiments (n = 3; *P < 0.05). ns: non-significant, A and B two different brands.

### 4.3. SEM of S. epidermidis Biofilm Development

**Figure 5.**
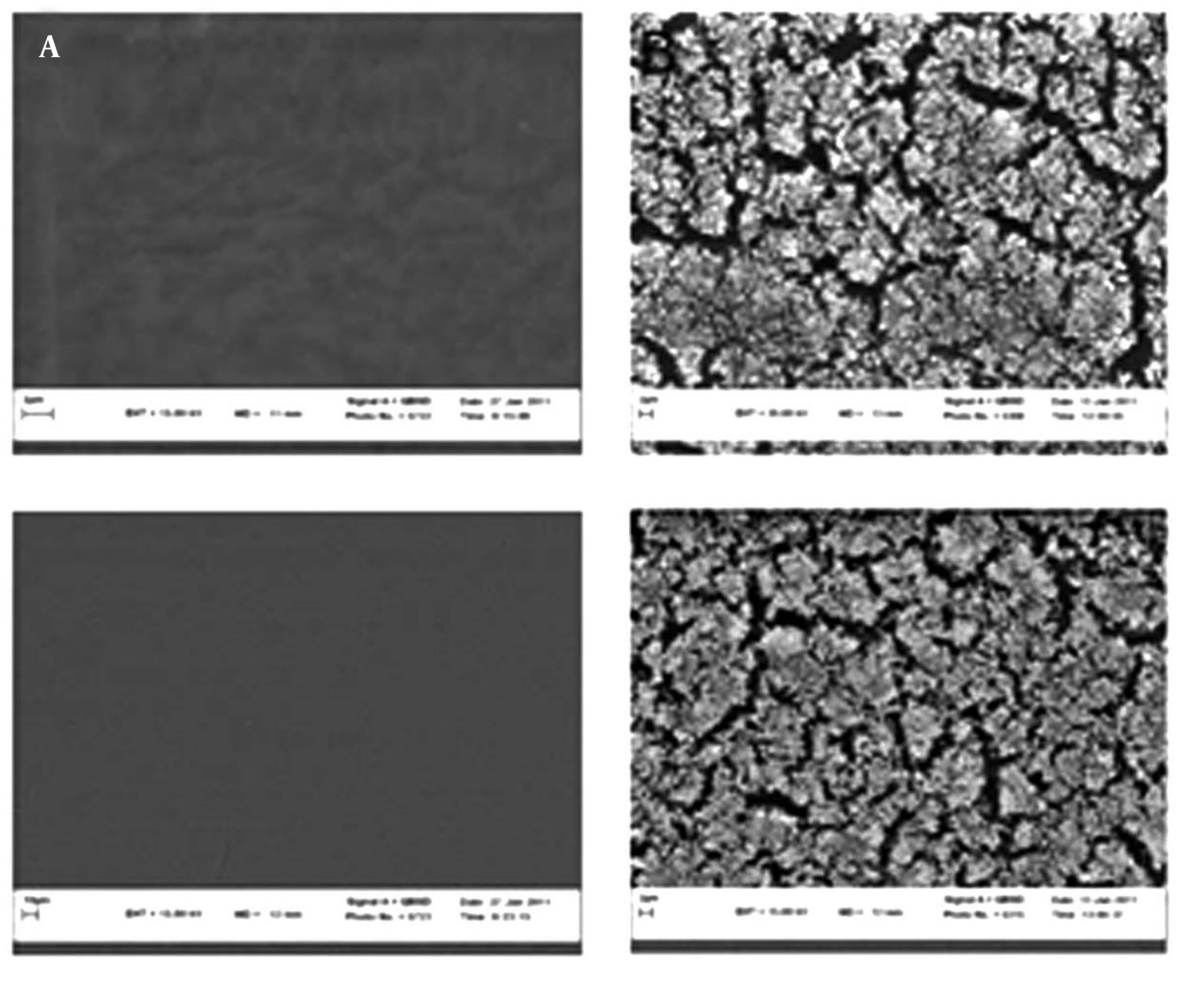
The Results of Scanning Electron Microscope (A) Control lense of A IOLs in ×10000, control lense of B IOLs in ×10000. (B) Biofilm formation by *S. epidermidis* (DSMZ 3270) on foldable IOL (1) Slime formation of bacteria on A IOL (field positive), Slime formation of bacteria on B IOL (field positive).

As observed in Figure 4, there were no significant differences between the two brands of hydrophilic acrylic at 24 and 72 hours with the DSMZ3270 strain (P > 0.05). However, there were no significant differences within each brand at 24 and 72 hours with the DSMZ3270 strain. There was no meaningful difference between hydrophilic and hydrophobic IOLs at 24 hours with the DSMZ3270 strain. Scanning electron microscopy (SEM) was performed to examine the biofilm and adherence of each IOL. Also four lenses were incubated with sterile TSB medium, as control, to evaluate surface properties of hydrophilic acrylic and hydrophobic PMMA lenses (Figure 5). Rates of biofilm-positive SEM fields on the IOLs were defined as the surface covered by biofilm on over at least half of the area.

A scanning electron microscope (SEM) was employed to observe the biofilm formation on each IOL material (Figure 5). The presence of biofilm was defined by recognition of slime and a multilayer formation of bacteria (Figure 5). In both brands of IOLs, the biofilm was recognized at 24 hours of incubation and developed over 48 hours.

## 5. Discussion

One of the most serious complications which arise after intraocular surgery is postoperative endophthalmitis. The attachment of bacteria to IOLs during implantation is the elementary phase in the pathogenesis of endophthalmitis and of pseudophakic occasional intraocular inflammations. Since 1949, when Ridley implanted the first poly made IOL (PMMA), there has been considerable progress in the applied designs and materials. The PMMA used in that first implantation has remained a popular material for IOL optics and is considered as the standard against which other materials are compared. Intraocular lens-associated biofilms have been the focal point of several recent reports, particularly in their relation with the assessment of staphylococcal biofilm formation on IOL materials. Three methods were performed including quantifying biofilm density by crystal violet staining and spectrophotometry, bacterial population enumeration, and finally the SEM of biofilm development.

Different conditions including the bacterial strains, the incubation time, IOL design, and the quantitative or qualitative methods determine bacterial adhesion. However, lens material (specially hydrophobicity or hydrophilicity) of the biomaterial stands as a basic variable condition in determining the bacterial adherence to the implant surfaces (15). According to the results of the current study, the bacteria and their products may tend to show less mucilaginous tendencies in adhering to the hydrophobic IOL materials than to the hydrophilic ones (Figures 1 and 2). Several researchers have attempted to determine the biomaterial which has the highest affinity for bacteria. The result of the present paper is in good harmony with the previous studies (1,2, 13). 

Meanwhile, the results of bacterial adhesion in some researches show that the adhesion of bacteria to the hydrophobic surfaces was greater than those of the hydrophilic ones (16). It indicates that the differences of the bacterial adhesion on various brands of IOL might be related to certain parameters such as hydrophobicity of both strain and IOL materials. The disparity between the results of the present study and those of other researchers could be related to these parameters. It means that the composition of each lens was different from the other lenses; therefore the amount of attachment was different. Cagavi et al. proved that hydrophobic coated IOLs decrease the bacterial colonization (17). The addition of heparin reduces the formation of biofilm on PMMA materials (18). 

The current study results suggest that the reduction of surface hydrophilicity hampers bacterial colonization. As observed in crystal violet assay, the bacterial population of hydrophilic acrylic lens was greater than those of the hydrophobic ones. These results correspond to the result of bacterial population enumeration assay. It was observed that by increasing the incubation time, the biofilm formation increased. The reason of this fact resides in the tendency of bacteria for adhesion on IOLs surface. These results were in accordance with those of the previous studies (19). It was also noteworthy that hydrophobic surface-modified IOLs lead to formation of fewer biofilms than the IOL, over hours. But in the case of hydrophilic lenses, the biofilm formation increased by escalating the incubation period. 

These data demonstrate that the attachment of bacteria to hydrophilic IOLs is more than their attachment to hydrophobic ones. Regarding these results, the bacterial strain might have more hydrophilic properties. As time passes, increasing the biomass of biofilm underlies the crucial role of time in biofilm formation on the IOL surfaces. The differences between IOL brands in the biofilm formation might state the influence of design parameters for IOLs. Biofilm formation is one of the several aspects related to post cataract surgery endophthalmitis. The current study showed that lens material and its hydrophilicity play an important role on the biofilm formation. The ideal biomaterial to prevent endophthalmitis does not yet exist. There is a need for further investigations to reduce the risk of endophthalmitis after cataract surgery.
